# Assessment of the Alga *Cladophora glomerata* as a Source for Cellulose Nanocrystals

**DOI:** 10.1021/acs.biomac.3c00380

**Published:** 2023-09-20

**Authors:** Karl Mihhels, Neptun Yousefi, Jaanika Blomster, Iina Solala, Laleh Solhi, Eero Kontturi

**Affiliations:** † Department of Bioproducts and Biosystems, Aalto-University, School of Chemical Engineering, 02150 Espoo, Finland; ‡ Ecosystems and Environment Research Program, Faculty of Biological and Environmental Sciences, University of Helsinki, 00014 Helsinki, Finland

## Abstract

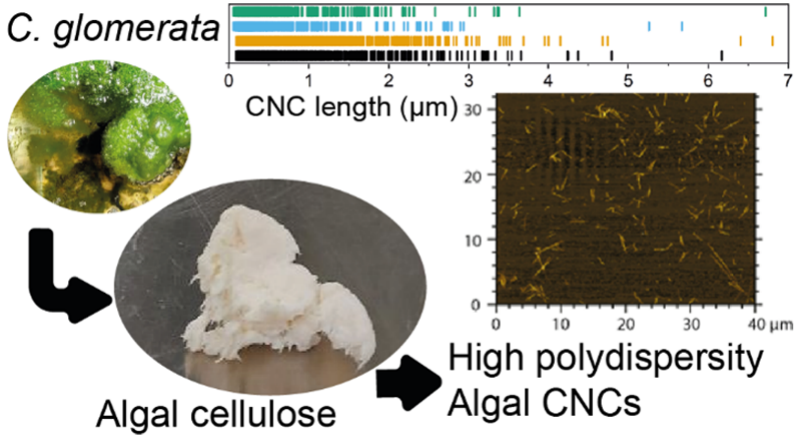

Nanocellulose is isolated from cellulosic fibers and exhibits many properties that macroscale cellulose lacks. Cellulose nanocrystals (CNCs) are a subcategory of nanocellulose made of stiff, rodlike, and highly crystalline nanoparticles. Algae of the order Cladophorales are the source of the longest cellulosic nanocrystals, but manufacturing these CNCs is not well-studied. So far, most publications have focused on the applications of this material, with the basic manufacturing parameters and material properties receiving little attention. In this article, we investigate the entirety of the current manufacturing process from raw algal biomass (*Cladophora glomerata*) to the isolation of algal cellulose nanocrystals. Yields and cellulose purities are investigated for algal cellulose and the relevant process intermediates. Furthermore, the effect of sulfuric acid hydrolysis, which is used to convert cellulose into CNCs and ultimately determines the material properties and some of the sustainability aspects, is examined and compared to literature results on wood cellulose nanocrystals. Long (>4 μm) CNCs form a small fraction of the overall number of CNCs but are still present in measurable amounts. The results define essential material properties for algal CNCs, simplifying their future use in functional cellulosic materials.

## 1 Introduction

Algae constitute a major source of biomass, which remains underutilized with respect to land plants, particularly in materials applications with cellulose. This negligence has continued even though the suitability of algal cellulose, the isolated cellulose fraction usually from green algae, has been demonstrated in a range of applications, such as energy storage,^1−3^ water and air purification,^4,5^ virus filtration,^6^ and tissue engineering scaffolding.^7^ From the processing standpoint, algal cellulose can be easily dispersed as a homogeneous hydrogel in water even after spray drying,^8,9^ unlike wood-based or bacterial cellulose.^8,9^

Further, a noteworthy issue is the distinct nature of nanocellulose that can be isolated from algal cellulose. Because of its high crystallinity,^10^ rodlike cellulose nanocrystals (CNCs) extracted from algal cellulose possess far higher aspect ratios than their counterparts from common biomass sources, such as wood, cotton, flax, or ramie.^11,12^ This is all the more important when recognizing the track record of long CNCs in composite applications in particular.^13,14^ Some of the most influential works^15−17^ on nanocellulose composites have been based on tunicate CNCs whose lengths far exceed those of the conventional sources from land plants. In many instances, algal CNCs have been reported to reach similar dimensions to those of tunicate CNCs, i.e., micron-sized lengths.^18−21^ However, there are very few studies that systematically explore algal cellulose as a source of CNCs, contrary to a wealth of detailed entries on the preparation of CNCs from wood pulp, cotton, or even tunicates.^22,23^ As green algae are among the major cellulose sources in the biosphere, their quality and potential as nanocellulose sources must be properly explored to gain a more comprehensive picture of the sustainability of nanocellulose.

In this work, we undertook a systematic study on the yield, chemical composition, and dimensions of CNCs prepared using the traditional sulfuric acid hydrolysis of algal cellulose isolated from *Cladophora glomerata*. It is the most widely distributed macroscopic alga throughout the freshwater ecosystem globally^24^ and the most dominant filamentous alga during summertime in the Baltic Sea.^25,26^ The development of the chemical composition along the whole route from raw biomass to algal cellulose was followed, enabling, among other issues, the evaluation of the true yield of the CNCs. Importantly, we could also demonstrate the full breadth of properties of the algal CNCs generated, namely, the exceptionally wide polydispersity in their length and the fact that the CNC properties were minorly affected by the acid hydrolysis conditions.

## 2 Experimental Details

### 2.1 Materials

The algae (*C. glomerata*) were kindly provided by the University of Helsinki (Zoological Station at Tvärminne, 2021-08-21). Acetic acid (glacial), NaOH (50% and 1 M), HCl (37%), H_2_SO_4_ (95–97%), and NaCl were supplied by VWR Chemicals. All VWR chemicals were purchased in analytical grade. NaClO_2_ (technical grade), arabinose, rhamnose, galactose, xylose, mannose, glucose (analytical grade), and TALH (titanium(IV) bis(ammonium-lactato)dihydroxide) were purchased from Sigma-Aldrich. PEI (poly(ether imide), branched, MW 70 000, 30%) was provided by Polysciences Inc. Cupri-ethylenediamine (CED) solution was purchased from FF-Chemicals. All chemicals were used as received. Polished silicon wafers were provided by OKMETIC. Ultrapure water (conductivity 0.8 μS/cm) from a MilliporeSynergy purification unit was used throughout the study.

### 2.2 Converting Raw Algal Biomass into Algal Cellulose

**Figure 1:**
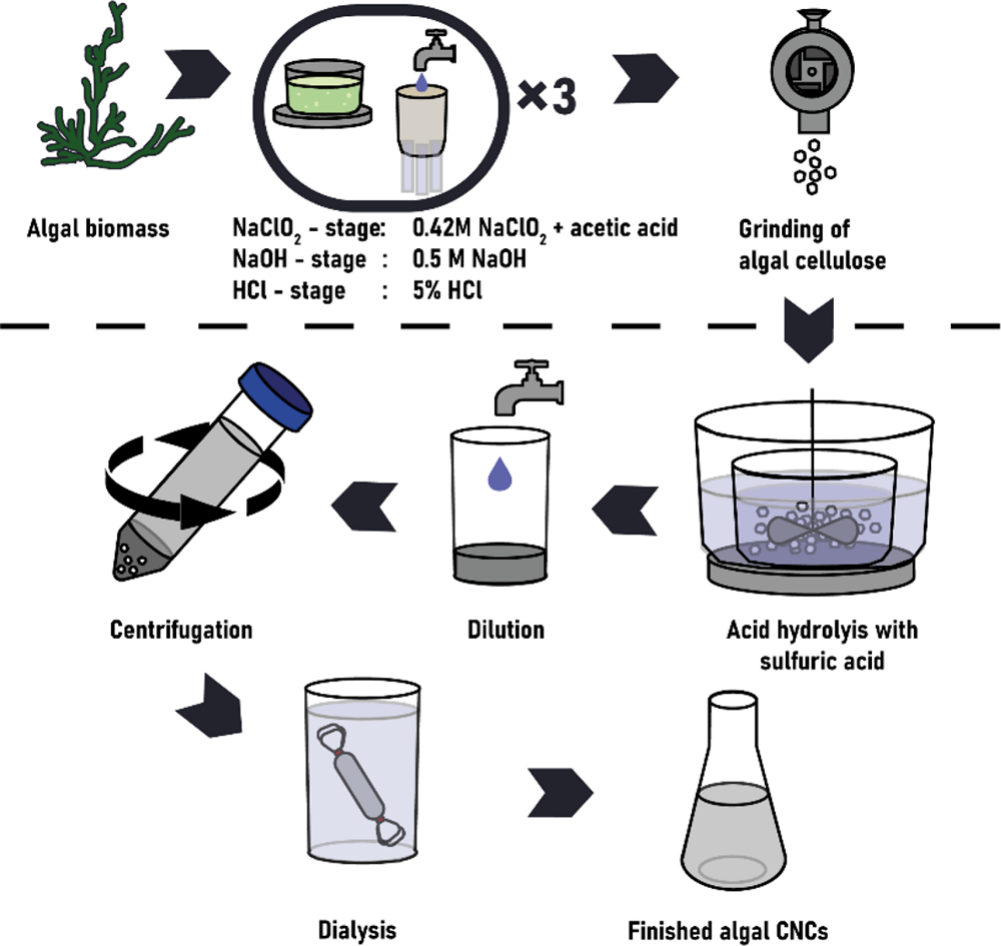
Pathway from raw algal biomass to algal cellulose nanocrystals.

Algal biomass was converted into algal cellulose using three consecutive chemical treatments (NaClO_2_ stage, NaOH stage, and HCl stage), followed by air drying and milling, as demonstrated in the top part of Figure 1.

This three-stage process was based on previous work by Mihranyan et al.^27^ In the first stage, the algal biomass was soaked in aqueous NaClO_2_ (0.42 M) and acetic acid (1.5%). During the second stage, the algal biomass was soaked in NaOH (0.5 M), and in the final stage it was soaked in HCl (5%). The algal biomass was washed between the stages using a 100 μm polyethylene sieve until the eluent was clear and neutral in pH. After the final washing, the solid residue (algal cellulose) was air dried and milled into a fine powder. More details on the preparation of algal cellulose from raw algae can be found in the Supporting Information (SI).

#### 2.2.1 Characterization of Algal Biomass, Reaction Intermediates, and Algal Cellulose

The yield, dry matter content^28^ (105 °C, 16 h), ash content^29^ (575 °C, 24 h), carbohydrate content^30^ (using Dionex Ultimate 3000 HPLC with a CarboPac PA20 column), and elemental composition (Thermo Flash Smart CHNSO Elemental Analyzer) of the raw algal biomass and the solid residue of each reaction stage were determined. The protein content of the algae was determined indirectly by multiplying the measured amount of nitrogen with an appropriate conversion factor (4.24).^31^ The limiting viscosity of algal cellulose dissolved in the CED solution was determined according to the standard ISO 5351:2004. To evaluate the degree of polymerization (DP) of the cellulose, a modified Mark–Houwink–Sakurada equation was used:^32^
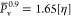
1where  represents the viscosity average DP and *η* is the intrinsic viscosity.

### 2.3 Converting Algal Cellulose into Cellulose Nanocrystals

Algal cellulose nanocrystals were prepared by sulfuric acid hydrolysis, and the preparation protocol, including the purification operations, is shown in the bottom part of Figure 1. The acid hydrolysis was conducted for four batches of algal cellulose by using the reaction conditions specified in Table 1. The experimental matrix was created by using a 3-factor, 2-level experimental design matrix (L4) according to the outlines set by Taguchi et al.^59^ The effects of each main parameter (temperature, acid concentration, and reaction time) were determined by averaging the results at a given level for each variable while treating the parameters not under study as experimental noise.

**Table 1:** Reaction Conditions during CNC Sulfuric Acid Hydrolysis

sample name	temperature (°C)	acid concentration^a^ (%)	reaction time (min)
T45_c64_t90	45	64	90
T45_c66_t45	45	66	45
T75_c64_t45	75	64	45
T75_c66_t90	75	66	90

a An acid-to-pulp ratio of 13.6 mL of acid/g of completely dry cellulose was used in all experiments.

After each reaction, the hydrolysis was stopped by diluting the reaction medium to 4% H_2_SO_4_. This diluted medium was then centrifuged multiple times with an Eppendorf 5804R centrifuge (15 557 rcf, 15 min) to collect the water-dispersible CNCs. The water-dispersible CNCs were further purified by dialysis using a SpectraPor7 (MWCO 3.5 kDa) membrane to a conductivity of <5 mS/m.

#### 2.3.1 Characterization of Algal Cellulose Nanocrystals

The surface charge of the cellulose nanocrystals was indirectly determined from the sulfur content obtained from elemental analysis. The particle size distributions of the CNCs were determined using atomic force microscopy (AFM, Bruker Multimode 8 AFM with MikroMasch HQ-NSC15/Al BS-tips). The CNCs were deposited as submonolayer films on silica wafers coated with TiO_2_ before measuring. The preparation procedures for the submonolayer films and TiO_2_ coating of the silicon wafer can be found in the SI. For each sample, a set of four images (40 × 40 μm, 1024 × 1024 points) was taken. The images were processed and the dimensions of the individual particles were analyzed using the MountainsSPIP EXPERT software (v.9.1). The limiting viscosity of the CNCs and the estimated DP from each sample were determined in a similar fashion as for algal cellulose after freeze-drying (Labconco Freezone 2.5) the CNC samples.

#### 2.3.2 Effects of Reaction Conditions during Acid Hydrolysis on CNCs and Their Properties

The effects of the reaction conditions (as shown in Table 1) were investigated using a Taguchi L4 matrix.^33^ The purpose of the experimental plan was to investigate the effects of temperature, sulfuric acid concentration, and time on the yields and resulting CNC properties such as surface charge, CNC length, CNC height, number/weight-average masses, polydispersity, and viscosity-average DP values. For calculating the number- and weight-average masses, the CNCs were assumed to have a square profile with a side length corresponding to the measured CNC height and a density of 1.61 g/cm^3^.^34^

The milder reaction conditions used matched the methods^35^ used for preparing wood CNCs (45 °C, 64% H_2_SO_4_, 25–45 min reaction time, acid-to-cellulose ratio of 8.75–17.5). The harsher reaction conditions (75 °C, 66% acid concentration, 90 min) were selected to investigate the claim^8^ that algal cellulose would be more resistant to chemical degradation than terrestrial sources of cellulose. The results were analyzed using a conventional one-way ANOVA.^36^

## 3 Results and Discussion

### 3.1 Characterization of Raw Algal Biomass, Process Intermediates, and Algal Cellulose

The chemical composition of the raw algal biomass, process intermediates, and algal cellulose is shown in Table 2. The major identified components of the raw algal biomass are cellulose (38.7%), ash (24.3%), heteropolysaccharides (13.7%), and proteins (6.2%). The sulfur content in the samples is most likely linked to sulfated heteropolysaccharides^37^ known to exist in this type of algae or sulfur-containing proteins. The relatively large mass of undetermined compounds (15.2%) can be attributed to a sum contribution of lipids, sulfated heteropolysaccharides, and unidentified metabolites and the underestimation of protein content.^37,38^

**Table 2:** Gradual Reduction of Impurities from Raw Algae to Algal Cellulose

	glucose (cellulose)	ash	heteropolysaccharides^b^	protein	sulfur	undetermined
raw algal biomass	38.7 ± 0.9%	24.3 ± 0.1%	13.7 ± 0.4%	6.2 ± 0.2%	2.0 ± 0.1%	15.2%
after the NaClO_2_ stage	89.1 ± 0.8%	2.9 ± 0.2%	1.0 ± 0.1%	1.9 ± 0.1%	0.1 ± 0.01%	5.0%
after the NaOH stage	98.0 ± 1.8%	2.7 ± 0.3%	0.9 ± 0.5%	0.9 ± 0.3%	N.D.^a^	2.5%^c^
after the HCl stage (algal cellulose)	99.4 ± 3.4%	0.6 ± 0.2%	0.4 ± 0.1%	0.1 ± 0.01%	N.D.^a^	0.5%^c^

a N.D.: content below the detection limit of the analysis.

b The heteropolysaccharides are a combination of arabinose, rhamnose, galactose, xylose, and mannose. The exact yields of these individual sugars can be found in the SI (Table S6).

c The total yield of the analyzed components exceeded 100% by this value. This is due to inaccuracies in the sample preparation or due to the overestimation of residual protein content.

When evaluating the progress of cellulose purification, most of the noncellulosic impurities are removed during the NaClO_2_ stage of the process. After the NaClO_2_ stage, the cellulose purity of the sample is already 89.1 ± 0.75%, and most of the hemicelluloses and proteins are removed. The decrease in ash content, however, occurs more likely due to a change in pH rather than reactions with NaClO_2_, as the largest component of ash in *C. glomerata* biomass is known to be CaCO_3_^39^ (66.7% of the total ash content). This CaCO_3_ will react with the acetic acid used in the NaClO_2_ stage via the well-known neutralization reaction mechanism.^40^
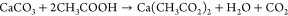
2The remaining ash removal happens due to the degeneration of the biomass structure and the leaching of other ash components during the washing.

At the NaOH stage, a cellulose purity of 98 ± 1.8% is reached. Most of the yield loss in cellulose (Table 3) takes place during this stage, and while the percentage content of other impurities changes very little between the NaClO_2_ stage and NaOH stage, the reduction in the absolute mass of these impurities is significant.The final glucose content of the algal cellulose after the HCl stage was 99.4 ± 3.4%, and the combined content of noncellulosic impurities was 1.1 ± 0.23%. This should be comparable to other studies in the field, where the purity is not reported by any quantitative method.^41−43^

**Table 3:** Increase in Glucose Purity and Change in Yield in the Processing Stages

	raw biomass	after the NaClO_2_ stage	after the NaOH stage	after the HCl stage
total yield (g)	100	39.8	16.5	15.4
glucose (cellulose) content (%)	38.7 ± 0.92	89.1 ± 0.75	98.0 ± 1.80	99.4 ± 3.4
glucose (cellulose) yield (g)	38.7 ± 0.92	35.5 ± 0.30	16.2 ± 0.29	15.3 ± 0.52
impurities yield (g)	61.3 ± 0.80	4.3 ± 0.16	0.3 ± 0.18	0.1 ± 0.05

Each processing stage simultaneously increases the purity but also decreases the yield of algal cellulose. In Table 3, the balance between the purity and the yield is demonstrated for 100 g of raw algal biomass. The NaClO_2_ stage produces a cellulosic material with purity comparable to wood-based, paper-grade pulps (73.3–91.6%).^44^ As wood-based, paper-grade pulps are the most common material for CNC production,^22^ the suitability of algal biomass for CNC production after this initial stage would warrant further study. The NaOH and HCl stages produce cellulose of higher purity but simultaneously inflict significant yield losses. These losses are most prominent during the NaOH stage, where more than half of the cellulose present in the biomass is lost. This loss is larger than expected, considering the mild conditions used for the alkaline pulping (0.5 M NaOH, 60 °C). A possible explanation for this is that the sample contained other glucose-containing compounds, such as starch or hemicelluloses containing glucose units, which were incorrectly classified as cellulose in the analytics. Ultimately, the application of the algal cellulose should dictate its preparation, as the application requirements for composites^45^ are very different from biomedical products, for example.^6^

### 3.2 Converting Algal Cellulose into Cellulose Nanocrystals

Table 4 reports the CNC yields and the amount of solid residue from each reaction. The CNC yields ranged from 32.7–72.6%, and the amount of solid residue from the reaction ranges from 1.3–15.0%. A small amount of solid residue suggests that the solubilization of algal cellulose is successful at most sample points.

**Table 4:** Yields of CNCs and Solid Residue on Both the Original Algal Biomass and the Algal Cellulose

parameters (temperature, acid concentration, and time)	CNC yield on raw biomass (%)	CNC yield on algal cellulose (%)	solid residue on raw algal biomass (%)	solid residue on algal cellulose (%)
T45_c64_t90	9.8 ± 0.3	67 ± 2	0.4 ± 0.1	2.8 ± 0.4
T45_c66_t45	4.7 ± 3	46 ± 19	0.4 ± 0.2	2.3 ± 1.3
T75_c64_t45	10.5 ± 1	67 ± 8	0.3 ± 0.1	1.9 ± 0.9
T75_c66_t90	5.4 ± 1	43 ± 8	1.5 ± 1.1	9.8 ± 7.4^a^

a The presence of black, insoluble residue (most likely humins^46,47^) was observed only in this sample.

To determine which parameters affected the yield of CNCs, we performed ANOVAs to determine the *p*-value of each parameter on each observed result. The smaller the *p*-value, the more likely it is that the observed result is due to the change in the studied parameter instead of the experimental noise. In our analysis, CNC yield was affected only by acid concentration (*p* = 0.14) with 64% H_2_SO_4_ corresponding to a yield of 67% and 66% H_2_SO_4_ corresponding to a yield of 45%, with reaction time (*p* = 0.94) and temperature (*p* = 0.96) having no apparent effect. This exceeds the recommended accuracy threshold of *p* = 0.05,^48^ but we believe it to be representative of the fact that the stability of the sulfuric acid hydrolysis as an experimental technique for producing algal CNCs has not been investigated in detail before. For all other parameters, the *p*-values exceeded a threshold of 0.15, and their ANOVAs are included in the SI.

In wood-based CNCs, using 64% H_2_SO_4_ is the practical upper limit for CNC production,^49^ and significant yield loss begins as the H_2_SO_4_ concentration exceeds 62%.^50^ The average algal CNC yield in our study (in 64% H_2_SO_4_) was 67% on algal cellulose (10.3% on raw algal biomass), while wood CNCs manufactured from cellulosic pulp with similar acid concentrations give yields of about 30%.^49^ As the typical yield of cellulose from kraft pulping is roughly 50%,^51^ the CNC yield on raw wood would be approximately 15%. This is relatively close to the CNC yields on raw algal biomass reported in this study and suggests that in yield, algal biomass could offer a competitive alternative for wood in CNC production. The best-reported yields of CNCs from wood pulp are ca. 66–69%,^52^ but it should be noted that these high yields were one-off studies where the quality of the CNCs was not scrutinized. If similar studies for manufacturing algal CNCs were carried out, similar or perhaps even larger increases in yield could be expected, particularly as algal cellulose is much more resistant to chemical degradation than wood cellulose.

### 3.3 Effects of Acid Hydrolysis on CNCs and Their Properties

**Figure 2:**
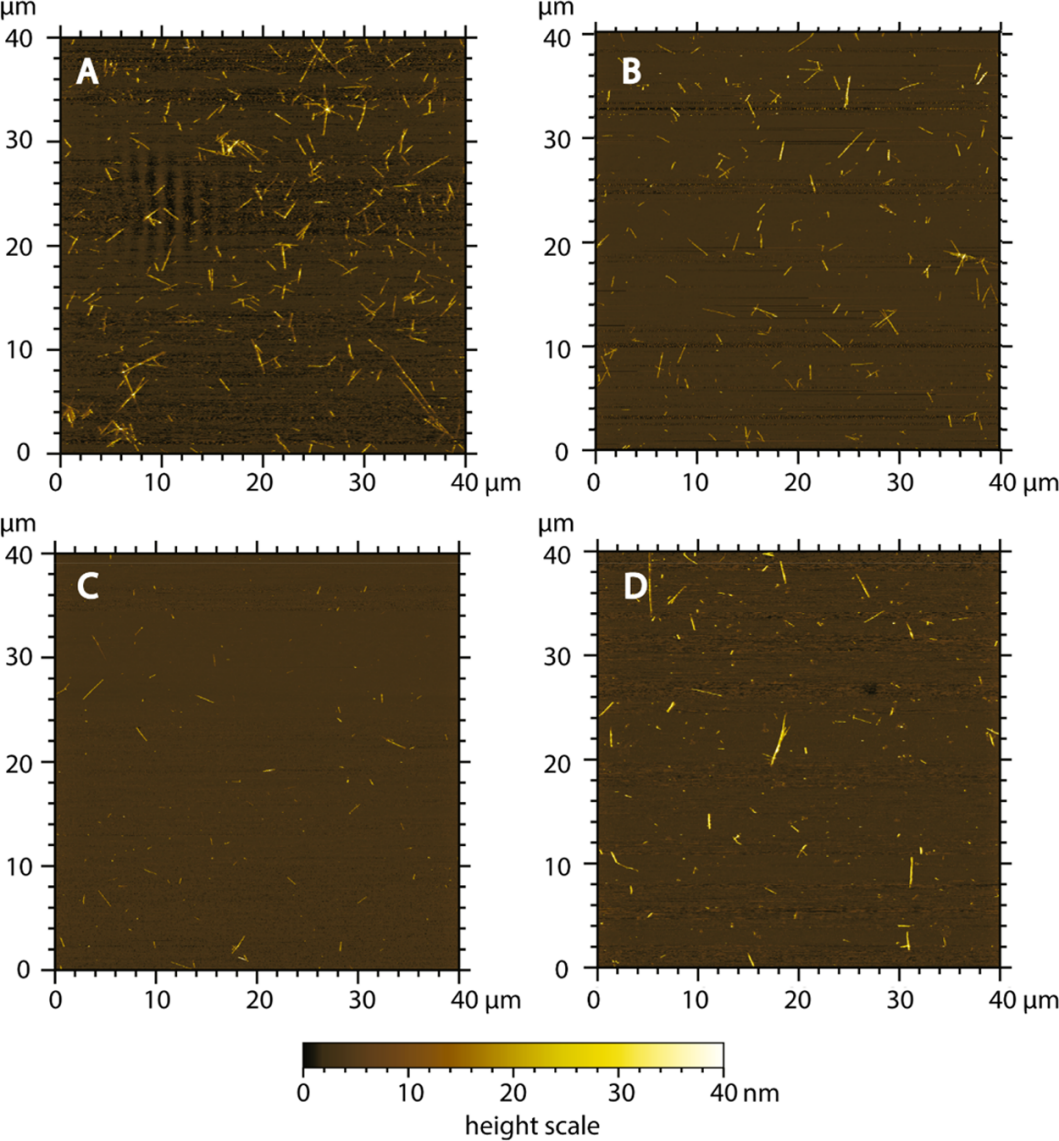
Representative AFM images of the CNCs under the following reaction conditions: (A) 45 °C, 64% acid, 90 min; (B) 45 °C, 66% acid, 45 min; (C) 75 °C, 64% acid, 45 min; and (D) 75 °C, 66% acid, 90 min.

**Figure 3:**
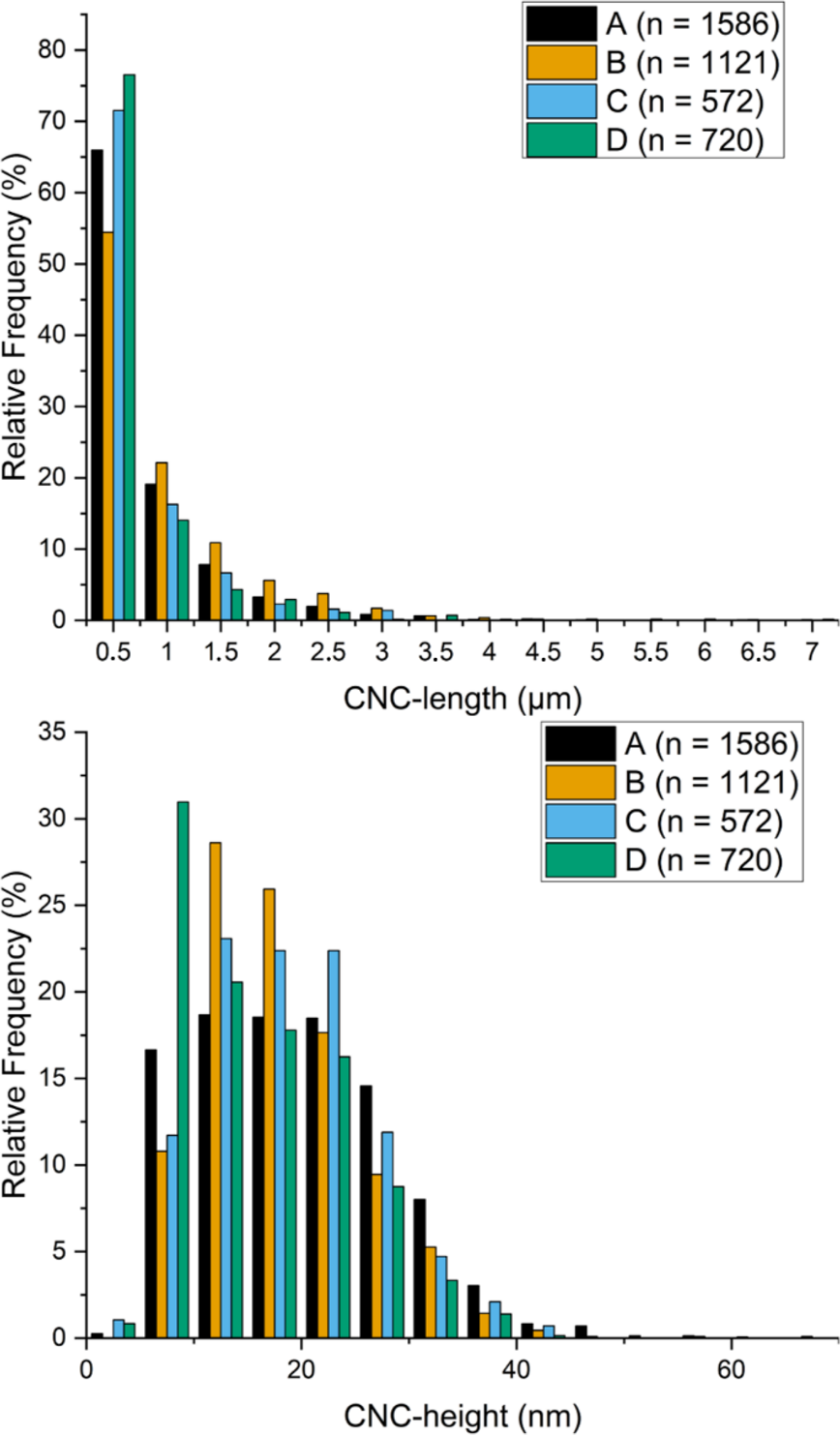
Size distributions of the CNCs: algal CNC lengths and algal CNC heights. The legends A–D refer to the reaction conditions, as in Figure 2. The distributions for the smaller (submicrometer) CNCs are shown in Figure S1.

Figure 2 shows the representative AFM images of each sample point. The average length of the CNCs was 540 ± 122 nm, and the average height/diameter was 18.0 ± 1.7 nm. The size distributions of the CNC lengths and heights, based on the full set of 16 AFM images, are illustrated in Figure 3.

No differences in CNC sizes could be determined from the ANOVA. The ANOVA of the results is included in Table S5. It is important to note that the extremely long CNCs (>4000 nm) are present in all samples, but only in a minority. It is equally important to note that even these longest CNCs bear a rodlike, rigid, and straight shape despite their abnormally high length, and they are therefore distinct from flexible cellulose nanofibers. The earliest reports on algal CNCs have most likely included some degree of sampling bias. The often-cited work by Kalashnikova et al.^18^ showed these extremely long CNCs at the surface of micellar structures instead of in water-dispersed medium. There is also evidence of possible error in the other direction. The relative amount of extremely long CNCs compared to the ones close to the average length is fairly small, and the studies where lower average lengths are reported for CNCs from species in the order Cladophorales^20,21^ might have used sample sizes too small to include the longest CNCs.

The sulfur content of the CNCs was 125 ± 17 mmol/kg, and the intrinsic CED viscosity was 431 ± 23. The intrinsic viscosity, viscosity average DP, and degree of sulfation of the algal CNCs have been compiled in Table 5 and compared to those of CNCs manufactured in similar reaction conditions (45 °C, 64% H_2_SO_4_, 10 min) from nonbleached softwood kraft-pulp.^53^ For the individual results of each sample point, see Table S2.Although CED viscometry can be uncertain with polydispersed samples,^54^ the DP of algal cellulose is markedly larger than that of wood cellulose. The same applies to CNCs made from these materials (Table 5).

**Table 5:** Viscosity Average DP and Degree of Sulfation of Algal CNCs and Wood CNCs

materials used in this study	intrinsic viscosity (*η*)	viscosity average DP	degree of sulfation (mmol/kg)
algal cellulose	1492 ± 82	5863	N.D.^a^
algal CNC (average)	431 ± 23	1476	125 ± 17
nonbleached softwood kraft-pulp^53^	674 ± 40	2424^b^	-
wood CNC^53^	61 ± 7	168^b^	296

a N.D.: content below the detection limit of the analysis.

b Recalculated using a more recent DP calculation method (1989)^32^ compared to the 1953^54^ method of the reference paper.^53^

The only confirmed result from the ANOVA of the AFM data and viscometry was that the reaction time influenced the CNC polydispersity (*p* = 0.04), with the average polydispersity being 4.65 at a 45 min reaction time and 5.46 at a 90 min reaction time. This conflicts with previous results on wood CNCs, where the increase in reaction time resulted in reduced polydispersity.^55^ In wood, this decrease in polydispersity was due to the loss of the longest CNCs in the material, while in algal cellulose, the long CNCs remain observable even at the harshest reaction conditions. The existence of very long CNCs can also explain the increase in the polydispersity of the material. Endwise degradation of cellulose crystals occurs during acid hydrolysis, and it takes place at the same rate for all CNCs. However, the decrease in length is more easily observed in the short CNCs, as the relative loss of length is larger for them. This phenomenon likely results in increased polydispersity. This is a major difference when compared to the behavior of wood CNCs, where even moderate increases in acid hydrolysis intensity are reflected by a clear reduction in the amount of the longest CNCs.^55^ For all the other studied CNC properties (length, height, surface charge, number-average mass, weight-average mass, and viscosity average DP), the respective *p*-values were in the range of 0.19–0.94, meaning that no effects of reaction parameters on CNC properties could be confirmed. The ANOVA for these results has been included in the Supporting Information.

Our main observation was that varying the reaction temperature, acid concentration, and time in a scale that produced observable differences in wood CNC lengths did not cause observable differences in algal CNC properties. We stress that although the actual data points for the CNC preparation conditions were scarce, they were chosen according to the Taguchi method. Altogether, this suggests that algal CNC crystallites have inherently superior chemical resistance properties compared to wood CNCs. Unlike in wood CNCs,^55^ the intensification of reaction conditions did not lead to the loss of the longest fractions of CNCs. However, the numerical amount of extremely long algal CNCs (>4000 nm) was comparatively small in all samples. Likely, both the presence of these extremely long crystallites and the high polydispersity observed in the material are inherent properties of algal cellulose manufactured by the current methods.

### 3.4 Sustainability of Algal CNCs

Although CNCs are green materials manufactured from renewable feedstocks, the environmental impact of their production has not yet been studied extensively.^22^ Part of the problem is that the best practices of CNC production have not yet been discovered. There is still a multitude of competing materials and production methods, each with its advantages and disadvantages regarding sustainability. The methods presented in this paper represent the state-of-the-art current practices of algal CNC manufacture.

What can be inferred from our results is the fact that the manufacturing methods for both algal cellulose and CNCs, while suitable for lab-scale work, do not make sense in larger-scale production and should be subjected to major revision. In the production of algal cellulose, unsustainable chemical dosages are used for cellulose purification (2.6 g of NaClO_2_, 0.97 mL of acetic acid, 1.3 g of NaOH, and 3.2 g of HCl for each 1 g of algal cellulose produced). Furthermore, the manufacturing process of algal cellulose is isolated from the process of making CNCs, and potential synergies, such as using the sulfuric acid process to at least partially replace the HCl stage of algal cellulose manufacture, are lost.

Regarding algal cellulose, the chemical dosages and the treatments used in the current purification methods match very closely with the methods developed by biologists in the 1950s.^60^ The goal of these biologists’ work was to identify cellulose as a structural component in various algal species, and their methods were aimed toward this purpose. The dosages and chemicals used reflect this original goal. The reason for the success of their method (and the slightly modified versions of it) in the current literature is most likely the fact that, as observed in our work, it produces highly pure algal cellulose. The issue of sustainability for algal CNCs has not been previously discussed in detail, and globally, the small scale of production of long CNCs (estimated to be <100 g/year in 2020)^61^ has allowed researchers to skirt this problem in the past.

Due to the aforementioned problems, we focus purely on the sustainability aspects of material properties of algal cellulose and algal CNCs, as they are likely to remain the same even if the production processes undergo changes and developments.

The main advantage of using algae as a CNC source is related to the inherent resistance of algal cellulose toward degradation. This study demonstrates that it boasts a higher resistance to degradation and dissolution by sulfuric acid than terrestrial celluloses. Dynamic thermogravimetric analysis of nanofibrillated algal cellulose has also shown that algal cellulose is thermally more stable than wood cellulose.^42^ This robustness of algal cellulose will be beneficial in most applications as the cellulose can undergo more intensive processing without loss in material properties.

Furthermore, terrestrial cultivation of most cellulose sources requires arable land, irrigation, and fertilizers.^56^ Algae, on the other hand, can be cultivated in almost any pool of water as the order Cladophorales contains both fresh- and saltwater-tolerant species, with *C. glomerata* being a freshwater species that can tolerate salinities up to 20‰.^57^ Furthermore, if harvested sustainably, then the use of *C. glomerata* biomass can have a positive environmental impact. As a fast-growing primary producer, *C. glomerata* binds considerable amounts of CO_2_ and nutrients (especially inorganic N) into its cells.^58^ This will considerably lighten the ecological burden of production when cradle-to-grave life cycle analyses are carried out.

## 4 Conclusions

The main observations of this study were (1) the current isolation process for algal cellulose and algal CNCs should be subjected to major revision, (2) the properties of algal CNCs are much less affected by acid hydrolysis conditions than terrestrial cellulose-based CNCs, and (3) the algal CNC mixture is extremely polydisperse, explaining the discrepancies in reported lengths in the literature. We prepared algal cellulose from the alga *C. glomerata* (purity 99.4%, yield 15.4%) with the conventional three-stage preparation process. It is questionable whether such purity is needed for most applications of this material. The first preparation stage already resulted in a cellulose purity of 89.1% with a yield of 38.7%. Therefore, the use of this process intermediate should be considered for applications in which purity requirements are secondary to structural properties and yields.

CNCs were prepared from algal cellulose using H_2_SO_4_ hydrolysis in various reaction conditions (temperature (45 or 75 °C), acid concentration (64% or 66%), and time (45 or 90 min)). The yield of the material was affected by the acid concentration, while varying the reaction time and temperature had little effect. In 64% H_2_SO_4_, we measured a 67% average yield of CNCs on algal cellulose (10.3% on raw algae), and in 66% H_2_SO_4_, we measured an average yield of 45% (6.9% on raw algae). The material properties of the CNCs remained practically identical in all reaction conditions. The average length of the produced CNCs was 540 ± 122 nm with a height of 18.0 ± 1.7 nm and a degree of sulfation of 125 ± 17 mmol/kg. The only observed material effect was a moderate increase in polydispersity (4.65 to 5.46) when the reaction time was increased from 45 to 90 min. The presence of extremely long CNCs (>4000 nm) was observed in all reaction conditions, albeit they were in the minority.

Our work has provided much-needed insights into the current preparation procedures for both algal cellulose and algal CNCs. The preparation of algal cellulose has not previously been studied in detail, and the respective effects of each preparation stage have remained unclear. It also is clear that wood and algae cellulose respond differently to sulfuric acid hydrolysis, yet currently the same protocols are used to make CNCs from both materials. The sustainability of algal CNC production is still hard to evaluate, but the higher chemical and thermal resistance of algal CNCs compared to wood CNCs allow for use in more applications, and the higher CNC length will also reflect positively upon final material properties. Finally, it should be noted that the raw material, *C. glomerata*, offers interesting opportunities not possible for terrestrial feedstocks regarding sustainable cultivation and harvesting.

## Supporting Information Available

Additional experimental details on the preparation of algal cellulose and the preparation of CNC submonolayer substrates for AFM analysis, a full report of the measured results at each sample point, the full ANOVA of these results, and the hemicellulose sugar profiles (PDF)
