# Supplementary material for: Assessment of the Alga Cladophora glomerata as a Source for Cellulose Nanocrystals
Source: Biomacromolecules. 2023 Sep 20;24(11):4672–9. doi: 10.1021/acs.biomac.3c00380 (PMC10646933; doi:10.1021/acs.biomac.3c00380)
Supplement: Supplementary file 1 — bm3c00380_si_001.pdf [file bm3c00380_si_001.pdf]

SUPPORTING INFORMATION

Assessment of the Alga *Cladophora glomerata* as a Source for Cellulose Nanocrystals

Karl Mihhels[§], *Neptun Yousefi*[§], *Jaanika Blomster*[ᵀ], *Iina Solala*[§], *Laleh Solhi*[§], *Eero Kontturi*[§]*

[§] Aalto University, School of Chemical Engineering, Department of Bioproducts and Biosystems, 02150, Espoo, Finland

[ᵀ] Ecosystems and Environment Research Program, Faculty of Biological and Environmental Sciences, University of Helsinki, 00014, Helsinki, Finland

Table of Contents

Additional Experimental Details……………………………………………..S2

ANOVA analysis………………………………………………………………..S4

Yields of the hemicellulosic sugars in manufacturing of algal cellulose….S8

Length distribution of CNCs with a bin width of 100nm………………….S9

Number of tables: 6

Number of pages: 9

Number of figures 1

1. Additional Experimental Details

Preparation of algal cellulose from the raw algal biomass

The algal cellulose used in the article was prepared using 100 g batches of air-dried Cladophora glomerata biomass. Each batch was subjected to the process stages detailed in Table S1. The eluent volumes were set to the levels specified here to ensure good mixing during the reactions, as smaller eluent usage resulted in only wetting the biomass and did not produce an aqueous media that could be mixed.

Supporting Table S1. Detailed reaction conditions of each stage

Process stage	Eluent	Reaction time	Reaction temperature
**$NaClO_2$-stage**	1000 ml 0.42 M $NaClO_2$ and 15 ml of glacial acetic acid	4 h	60 °C
**NaOH-stage**	1000 ml 0.5 M NaOH	16 h	60 °C
**HCl-stage**	1000 ml 5 m-% HCl	16 h	Heated momentarily to 85 °C and left to cool afterwards in room temperature

Preparation of TiO_2-coated submonolayers

For measuring the size distribution samples were prepared by spin coating submonolayers of CNCs on a polished silica wafer. The exact procedure used here is described, as proper preparation

of submonolayer films of cellulose for AFM-imaging is not trivial, especially for large AFM-images where local surface imperfections may cause damage to the tip and corrupt the data collection for the entire image. The following preparation protocol allowed for consistent production 40 μm × 40 μm AFM-images with good quality and was seen as a valuable addition to the work. The following procedure is expected to work for all negatively charged CNCs, as the adhesion between the CNCs and the TiO_2 surface is electrostatic in nature.

The preparation of the silica wafer coating deviated slightly from the method used by Kontturi et. al.[1] Instead of spin coating TALH (titanium(IV)-bis-(ammonium-lactato)-dihydroxide) solution directly on a polished silica wafer. a layer-by-layer coating inspired by Shi et. al.[2] proved better at providing a smooth background for the subsequent AFM-imaging. To prepare the surface for spin coating with CNCs, polished silicon wafers were first treated with the RCA-1 cleaning protocol[3] and rinsed with water. After this, they were placed into a 0.5 M NaCl. 1g/l PEI-solution (Branched, Mw 70,000) for 15 minutes and rinsed with water, and finally immersed into a 5% TALH-solution for 15 minutes and rinsed with water. After preparing the surface, the wafers were held overnight in an oven at 450 °C to convert the TALH into TiO_2. Then, the wafers were cut to approximately 5 mm × 5 mm squares (to fit the spin coating and AFM-imaging apparatus) and these squares were cleaned with pressurized air to remove any residual wafer dust resulting from the cutting. Prior to spin coating, the TiO_2 surface of the wafer was exposed to a UV-Ozone cleaner (Biofore nanosciences) for 10 min.The CNC solutions were diluted to 60 mg/l prior to coating and spin coated at 4000 rpm.

2. ANOVA-analysis

In ANOVA, each variable was analysed in respect to each parameter variable pair, which is presented in Supporting Table S2.

Supporting Table S2. Results from the sample points

**Test point**	**T45_c64_t90**	**T45_c66_t45**	**T75_c64_t45**	**T75_c66_t90**
**Parameters**				
**Temperature**	45	45	75	75
Time	90	45	45	90
H2SO4 concentration	64	66	64	66
**Variables**				
Average length (nm)	535	709	487	425
Average height (nm)	19.5	18.3	18.5	15.6
Number average mass (g)	5.27E-17	5.08E-17	4.19E-17	2.84E-17
Weight average mass (g)	2.94E-16	2.39E-16	1.93E-16	1.52E-16
Polydispersity index	5.57	4.69	4.60	5.35
Sulfur content (m%)	0.33%	0.51%	0.36%	0.40%
Yield	68%	33%	73%	37%
Yield of solid residuals	3.2%	3.1%	1.2%	15.0%
**CED-viscosity (η)**	468±6	437±3	434±4	386±1
**DP_v**	1617	1498	1488	1305

The analysis proceeded as follows (Using yield analysis for temperature as an example):

First, the parameter pairs were identified. For temperature the pairs were (T45_c64_t45 and T45_c66_t45), corresponding to low temperature and (T75_c64_t45 and T75_c66_t90) corresponding to high temperature. This set the k for the ANOVA-analysis to 2 (k = number of populations or treatments being compared). Respectively the n was 4. (n = total number of samples in the experiment).

Then the grand mean was calculated using the following formula:

$$\overline{y}_{\text{grand}} = \frac{y_n}{n} = \frac{y_1 + y_2 + y_3 + y_4}{n} = \frac{67.9\% + 32.7\% + 72.6\% + 37.4\%}{4} = 52.6\%$$

And the means of high and low temperature pairs were calculated

$$\overline{y}_{high} = \frac{y_{high}}{n_{high}} = \frac{72.6\% + 37.4\%}{2} = 55.0\%$$

$$\overline{y}_{\text{low}} = \frac{y_{\text{low}}}{n_{\text{low}}} = \frac{67.9\% + 32.7\%}{2} = 50.3\%$$

From this the sum of square of treatments (SSTr) could be calculated using the formula

$$SSTr = \sum \left(\overline{y}_{grand} - \overline{y}_k\right)^2 = \left(\overline{y}_{grand} - \overline{y}_{high}\right)^2 + \left(\overline{y}_{grand} - \overline{y}_{low}\right)^2$$
$$= (52.6 - 55.0)^2 + (52.6 - 50.3)^2 = 0.0011 \ldots$$

And the sum of square of error (SSE) using the formula

$$\text{SSE} = \sum \left(\overline{y}_{\text{low}} - \overline{y}_{1,2}\right)^2 + \sum \left(\overline{y}_{\text{high}} - \overline{y}_{3,4}\right)^2$$

$$= \left(\overline{y}_{\text{low}} - y_1\right)^2 + \left(\overline{y}_{\text{low}} - y_2\right)^2 + \left(\overline{y}_{\text{high}} - y_3\right)^2 + \left(\overline{y}_{\text{high}} - y_4\right)^2$$

$$= (50.3 - 67.9)^2 + (50.3 - 32.7)^2 + (55.0 - 72.6)^2 + (55.0 - 37.4)^2 = 0.12 \ldots$$

And finally, the sum of squares total (SST) using the formula

$$SST = SSTr + SSE = 0.0011 \ldots + 0.12 \ldots = 0.13 \ldots$$

Then, the ANOVA-table was filled according to the template presented in Supporting Table S3. The example of yield analysis for temperature can be seen in Supporting Table S4.

Supporting Table S3. ANOVA-analysis template

Source of Variation	Degree of freedom	Sum of Squares	Mean Square	F-Ratio	p-value
**Treatment**	k - 1	SSTr	$MSTr = \frac{SST}{k-1}$	$\frac{MSTr}{MSE}$	$F.DIST.RT(\frac{MSTr}{MSE}, k-1, n-k)^{a}$
**Error**	n - k	SSE	$MSE = \frac{SSE}{n-k}$		
**Total**	n - 1	SST			

[a]The F.DIST.RT-function is a Microsoft Excel function that returns the (right-tailed) F probability distribution (degree of diversity) for two data sets.

Supporting Table S4. Anova analysis of the Yield-Temperature pair

Source of Variation	Degree of freedom	Sum of Squares	Mean Square	F-Ratio	p-value
**Treatment**	1	0.0011…	$\frac{0.0011\ldots}{1} = 0.0011\ldots$	$\frac{0.0011\ldots}{0.06\ldots}$	0.91…
**Error**	2	0.12…	$\frac{0.12\ldots}{2} = 0.06\ldots$		
**Total**	3	0.13…			

The analysis proceeded similarly for all other parameter-variable combinations, producing the following p-values. The cut-off limit for p-values was set to 0.05, corresponding to a 5% risk in concluding that a difference exists when there is no actual difference. All the calculated p-values are shown Table S5.

Supporting Table S5. p-value test results

Variable	Parameter	p-value	p-value test at 0.05 passed
**Average length (nm)**	Temperature	0.33	NO
**Average length (nm)**	Time	0.57	NO
**Average length (nm)**	Acid concentration	0.81	NO
**Average height (nm)**	Temperature	0.50	NO
**Average height (nm)**	Time	0.80	NO
**Average height (nm)**	Acid concentration	0.41	NO
**Number average mass (g)**	Temperature	0.23	NO
**Number average mass (g)**	Time	0.78	NO
**Number average mass (g)**	Acid concentration	0.70	NO
**Weight average mass (g)**	Temperature	0.19	NO
**Weight average mass (g)**	Time	0.95	NO
**Weight average mass (g)**	Acid concentration	0.66	NO
**Polydispersity index**	Temperature	0.86	NO
**Polydispersity index**	Time	0.04	YES
**Polydispersity index**	Acid concentration	0.94	NO
**Sulfur content (m-%)**	Temperature	0.77	NO
**Sulfur content (m-%)**	Time	0.61	NO

**Sulfur content (m-%)**	Acid concentration	0.31	NO
**Yield on algal cellulose (%)**	Temperature	0.91	NO
**Yield on algal cellulose (%)**	Time	1.00	NO
**Yield on algal cellulose (%)**	Acid concentration	0.02	YES
**Yield of solid residuals**	Temperature	0.62	NO
**Yield of solid residuals**	Time	0.50	NO
**Yield of solid residuals**	Acid concentration	0.28	NO
**Viscometry (η)**	Temperature	0.41	NO
**Viscometry (η)**	Time	0.67	NO
**Viscometry (η)**	Acid concentration	0.67	NO

3. Yields of the hemicellulosic sugars in manufacturing of algal cellulose

Supporting Table S6. Yields of the hemicellulosic sugars in manufacturing of algal cellulose

**Processing stage**	**Arabinose**	**Rhamnose**	**Galactose**	**Xylose**	**Mannose**
**Raw algal biomass**	6.20% ± 0.15%	0.48% ± 0.01%	4.29% ± 0.11%	2.25% ± 0.04%	0.44% ± 0.11%
**After $NaClO_2$-stage**	0.40% ± 0.02%	N.D.	0.50% ± 0.01%	0.19% ± 0.01%	N.D.
**After NaOH-stage**	0.26% ± 0.01%	0.05% ± 0.07%	0.28% ± 0.01%	0.09% ± 0.12%	0.20% ± 0.28%
**After HCl-stage (algal cellulose)**	N.D.	N.D.	0.11% ± 0.01%	0.32% ± 0.02%	N.D.

N.D. : content below the detection limit of the analysis

4. Length distribution of CNCs with a bin width of 100 nm

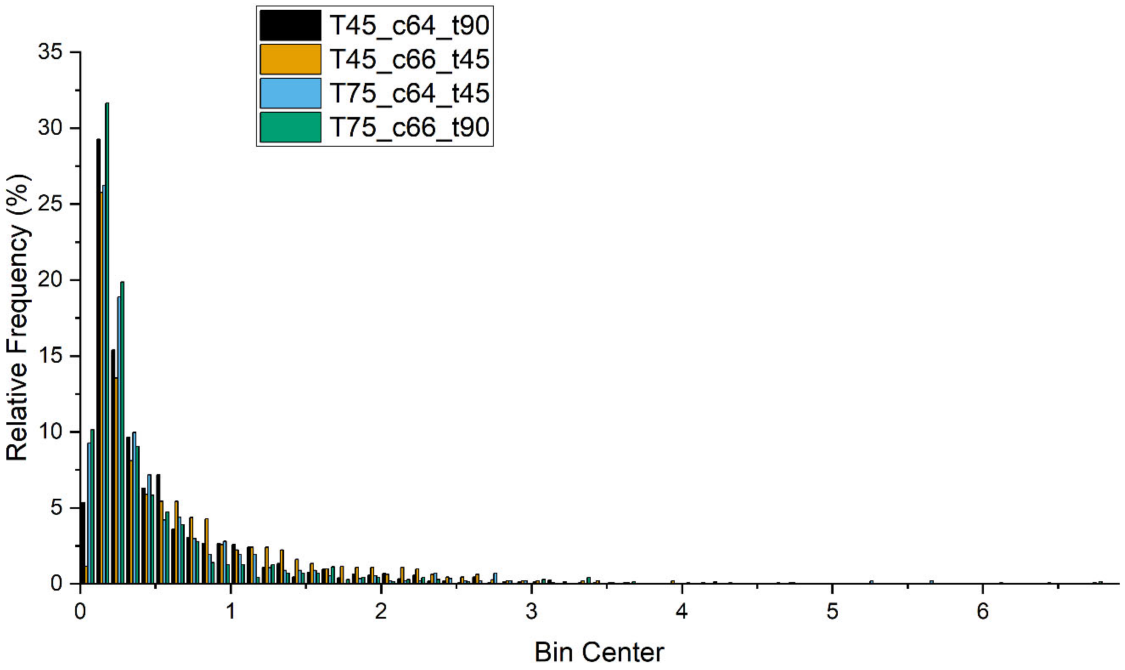

Figure S1. Length distribution of CNCs with a bin width of 100 nm.

References

(1) Kontturi, E.; Johansson, L. S.; Kontturi, K. S.; Ahonen, P.; Thüne, P. C.; Laine, J. Cellulose nanocrystal submonolayers by spin coating. *Langmuir* **2007**, *23*, 9674-9680. DOI: 10.1021/la701262x

(2) Shi, X.; and Cassagneau, T.; Caruso, F. Electrostatic interactions between polyelectrolytes and a titania precursor: Thin film and solution studies. *Langmuir* **2002**, *18*, 904-910. DOI: 10.1021/la011310d

(3) Bachman, M. *Cleaning Procedures for Silicon Wafers*; University of California, Irvine, Irvine, CA, 2002. https://www.inrf.uci.edu/wordpress/wp-content/uploads/sop-wet-silicon-rca-1.pdf (Accessed 2023-02-02)
